# The Relationship between Stasis-Stagnation Constitution and Peripheral Arterial Disease in Patients with Type 2 Diabetes

**DOI:** 10.1155/2014/903798

**Published:** 2014-02-11

**Authors:** Kuo-Chin Huang, Ching-Chu Chen, Yi-Chang Su, Jui-Shan Lin, Chwen-Tzuei Chang, Tzu-Yuan Wang, Rong-Hsing Chen, Fuu-Jen Tsai

**Affiliations:** ^1^Graduate Institute of Chinese Medicine, China Medical University, Taichung 40402, Taiwan; ^2^Department of Integration of Traditional Chinese and Western Medicine, China Medical University Hospital, Taichung 40447, Taiwan; ^3^School of Chinese Medicine, College of Chinese Medicine, China Medical University, Taichung 40402, Taiwan; ^4^Division of Endocrinology and Metabolism, Department of Medicine, China Medical University Hospital, Taichung 40447, Taiwan; ^5^Department of Medical Genetics, Medical Research and Pediatrics, China Medical University Hospital, Taichung 40447, Taiwan; ^6^Department of Biotechnology, Asia University, Taichung 41354, Taiwan

## Abstract

*Objectives*. In traditional Chinese medicine, Yu-Zhi (YZ, indicating stasis and stagnation) constitution describes a body that tends to express abnormal circulatory conditions. This study identified the linkage between YZ constitution and peripheral arterial disease (PAD) in patients with type 2 diabetes. *Methods*. Patients over 20 years of age who had had type 2 diabetes for 5 years or longer were recruited. PAD was diagnosed if the ankle-brachial index score was ≤0.9 in either leg. Level of YZ constitution was accessed by an YZ Constitution Questionnaire. *Results*. A total of 712 patients (354 men and 358 women) with a mean age of 61.5 ± 10.6 years and diabetes duration of 13.1 ± 6.7 years were recruited. The prevalence of PAD among our patients was 7.2%. Multivariate logistic regression revealed significant correlations between PAD and, respectively, YZ score, age, diabetes duration, current smoking, and hs-CRP. *Conclusion*. In addition to traditional risk factors, YZ constitution was statistically associated with PAD in patients with type 2 diabetes. This result invites further research into the effectiveness of traditional Chinese medicine to treat YZ constitution.

## 1. Introduction

Atherosclerosis is a leading cause of mortality and morbidity in the developed world. Peripheral arterial disease (PAD), one clinical manifestation of atherosclerosis, is an independent risk factor of mortality and morbidity in patients with cardiovascular diseases [1]. The complications of PAD, including foot ulceration and gangrene, increase the likelihood that the patient may require lower extremity amputation [2], with a resulting mobility loss and disability. PAD is a macrovascular complication of diabetes mellitus (DM). The high prevalence of PAD in diabetes patients is a reason that DM has become the leading cause of nontraumatic lower-limb amputation in developed countries [3]. Although several risk factors for PAD have been identified, physicians and patients usually are unaware of the condition until symptoms occur [4]. The identification of high risk for PAD in diabetes patients might provide an opportunity for early protection and treatment, thus preventing progression of PAD. However, at present, no such method for early identification exists.

Constitutional diagnosis has been used for more than 2000 years by traditional Chinese medicine (TCM) to classify patients. According to a textbook of TCM internal medicine, *Huang Di Nei Jing*, specific constitutions develop from both congenital and environment factors. The patient's constitution affects his or her susceptibility to and the development and prognosis of a specific disease. The classification method in TCM differs from the clinical practice of conventional Western medicine. Thus, different TCM constitutions may be encountered in patients diagnosed according to Western practices as having the same disease.

The Yu-Zhi (YZ) constitution is one of the TCM body constitutions and represents a tendency to stasis or stagnation. It tends to express blood stasis syndrome (BSS), a morbid state caused by blood stagnation, which is considered a link to cardiovascular disturbance. One study found that BSS was associated with increased thickness of the carotid intima-media in dyslipidemia patients [5]. In TCM, BSS constitutes the main pathological component for cardiovascular diseases and cardiovascular complications of DM [6–8]. Intuitively, the YZ constitution is typically regarded as correlating with cardiovascular events, but the empirical relationship between YZ constitution and PAD caused by diabetes remains unclear. The current study used a cross-sectional design to examine the possible association between YZ constitution and PAD in patients with type 2 diabetes.

## 2. Patients and Methods

### 2.1. Study Subjects

Nonaboriginal Taiwanese type 2 diabetes patients were recruited at the outpatient clinic of China Medical University Hospital between September 2006 and June 2007. All study patients were older than 20 years and had had DM for 5 years or longer. Type 2 diabetes was diagnosed according to the American Diabetic Association Criteria [9]. Ankle-brachial index (ABI) was used to diagnose PAD. Patients with type 1, gestational, or maturity-onset diabetes of the young were excluded. This research was approved by the Institutional Review Board of China Medical University Hospital, and all participants provided informed consent.

### 2.2. Data Collection

Questionnaire collection, ABI measurement, and data management were performed by various personnel. Demographic data (age, sex, age at diagnosis of diabetes, and smoking history) were collected by a questionnaire. Systolic, and diastolic blood pressure (SBP and DBP) were obtained by averaging 2 measurements taken at least 5 min apart, with an interval of rest in between. The participants' body height and weight (while wearing light clothing and no shoes) were measured by experienced research staff, and the body mass index (BMI) was calculated as a ratio of body weight (kg) divided by the square of body height (m^2^). After patients had fasted overnight, their blood samples were drawn between 8:00 and 10:00 in the morning, and the separated serum was stored at −70°C until assaying. Fasting plasma glucose (FPG) was determined by the hexokinase method (Integra 700, Roche, Mannheim, Germany). Hemoglobin A1c was measured using high-performance liquid chromatography (HLC-723G7, TOSOH Bioscience, Tokyo, Japan). Serum levels of total cholesterol (TC), triglyceride, high- (HDL) and low-density lipoprotein (LDL) cholesterol, creatinine, and uric acid were examined by standard laboratory methods (Integra 700, Roche). High-sensitivity C-reactive protein (hs-CRP) was gauged by immunoturbidimetry (Integra 700, Roche). The albumin-to-creatinine ratio (ACR) was obtained from morning spot urine samples, and data were categorized as normoalbuminuria (ACR ≤ 30 mg/g), microalbuminuria (30 < ACR < 300 mg/g), or macroalbuminuria (ACR ≥ 300 mg/g). Biochemistry analysis was conducted in the Taipei Institute of Pathology.

### 2.3. Measurement of ABI

Ankle-brachial index was measured by the Colin noninvasive vascular screening device (model BP-203 RPE III, Colin Medical Technology, Komaki, Japan). After patients had rested for 5 min in the supine position, their blood pressure was measured from bilateral arm and ankle, and pulse volume was measured from the brachial and posterior tibial arteries. The highest arm pressure was used to calculate an ABI score. PAD was diagnosed if the ABI score (which is the lowest ratio of ankle-to-arm SBP) was equal to or less than 0.90 [10]. Patients with ABI > 1.3 were excluded from the study, because they might have incompressible calcified arteries, which would cause misclassification [11].

### 2.4. Yu-Zhi Constitution Questionnaire

The questionnaire used to assess YZ body constitution contains 8 items (see the appendix) and was originally developed through a psychometrically sound method [12]. Its internal consistency reliability has been measured and yielded a Cronbach's *α* of 0.7827, which is considered acceptable for clinical use. Validity of the instrument was measured by assessing its construct validity. Two principal components explained 54.7% of the total variance. The 8 questionnaire items probed self-reported symptoms, with responses being rated on a 5-point Likert scale (never, occasionally, sometimes, often, and always). The final YZ score was obtained by summing the scores for all items, with higher scores indicating stronger intensity of YZ constitution.

### 2.5. Statistical Analysis

Continuous variables were expressed as mean ± standard deviation. Categorical variables were expressed as percentages. Differences in the results for type 2 diabetes patients with or without PAD were compared by Student's *t*-test for continuous variables and by *χ* ^2^ test for categorical variables. The association between YZ score and PAD was assessed by the logistic regression model to calculate the odds ratios (OR) and their 95% confidence intervals (CIs). All analyses were performed using SPSS 12.0 software (SPSS Inc., Chicago, IL), and *P* values of less than 0.05 were considered statistically significant.

## 3. Results

In all, 718 unrelated people were recruited. Six people were excluded because of an ABI > 1.3, yielding a final study group of 712 patients with type 2 diabetes (354 men and 358 women). The demographic and anthropometric traits as well as plasma and urine biochemical data of the patients are presented in Table 1. The study group had a mean age of 61.5 ± 10.6 years and a mean diabetes duration of 13.1 ± 6.7 years. The PAD prevalence rate among the participants was 7.2%.

Table 1 shows that patients with PAD attained significantly higher YZ scores than those without PAD (*P* = 0.006). In addition, compared with the non-PAD group, the PAD patients were significantly older (*P* = 0.003), had experienced a longer duration of diabetes (*P* = 0.008), and included more current smokers (*P* = 0.033). The PAD group displayed a significantly higher SBP (*P* = 0.002), higher hs-CRP (*P* = 0.049), higher creatinine (*P* = 0.016), higher uric acid (*P* = 0.004), and higher proportions of micro- and macroalbuminuria (*P* < 0.001). Sex, DBP, body height, body weight, BMI, FPG, hemoglobin A1c, TC, triglycerides, HDL cholesterol, and LDL cholesterol did not significantly differ between the PAD and non-PAD group.

Table 2 shows the adjusted and unadjusted logistic regression plots correlating key variables with PAD. For the crude ORs, the variables of age, diabetes duration, current smoking, SBP, hs-CRP, uric acid, ACR, and YZ score were linked with PAD. In the age- and sex-adjusted analysis, the variables of diabetes duration, current smoking, SBP, BMI, TC, LDL cholesterol, hs-CRP, uric acid, ACR, and YZ score were associated with PAD. A full model using multivariate logistic regression showed that the independent association factors were age (OR 1.04, 95% CI 1.00–1.07), diabetes duration (OR 1.06, 95% CI 1.02–1.10), current smoking (OR 3.14, 95% CI 1.33–7.39), hs-CRP (OR 1.29, 95% CI 1.08–1.54), and YZ score (OR 1.10, 95% CI 1.00–1.21).

## 4. Discussion

The results of this cross-sectional study revealed that YZ constitution was a predictor for PAD in type 2 diabetes patients. This finding indicated a relationship between YZ constitution and cardiovascular complications of type 2 diabetes. Based on results of this study, type 2 diabetes patients who exhibit symptoms and signs of YZ constitution, including limb numbness; chest tightness; tingling pain; bruises without an apparent cause; dry, cracked, scaly, or tough skin; dull and lustreless face, and dull purple color or petechiae on the lips or tongue, might have a higher risk of PAD.

People with a YZ constitution tend to express BSS, which is a morbid state of blood stagnation usually considered relevant to cardiovascular complications. Prior researches found that BSS was related to increased carotid intima-media thickness in dyslipidemia patients [5]. The severity of BSS also correlated with an index of chronic renal pathology; moreover, urine protein, cholesterol, triglyceride, and renal function were positively correlated with the severity of BSS [13]. However, these studies did not distinguish between the effects of BSS versus traditional cardiovascular risk factors. We adjusted for traditional cardiovascular risk factors using multivariate logistic regression, and our results indicated that YZ constitution showed an independent association with PAD in patients with type 2 diabetes. For the PAD is a clinical manifestation of atherosclerosis, results of the present study gave an idea that YZ constitution promotes the formation of atherosclerosis through mechanisms that are different from traditional cardiovascular risk factors.

Further investigation is needed to ascertain how YZ constitution influences PAD development in type 2 diabetes. The mechanisms related to the formation of YZ constitution are known to be complex. Associated pathogenesis of BSS includes microcirculation disturbance, hemodynamic change, and abnormal hemorheologic factors such as eicosanoid-controlled functions; fibronectin; the fluidity of human erythrocyte membranes, and plate activation, aggregation, and adhesion. Microcirculation disturbance is also considered fundamental to cardiovascular diseases [14]. In DM patients, platelet activation marker [15] and endothelium dysfunction [16] are strongly associated with BSS. Among these various factors, microcirculation disturbance and hemorheologic factors correlate with other traditional cardiovascular risk factors [17]. Moreover, in TCM, congenital factors are considered to be a principal component of a person's constitution. Thus, whether the YZ constitution has a genetic basis is a topic of scientific interest.

Traditionally, the therapeutic methods of TCM for the treatment of BSS are termed “promoting blood circulation to remove stasis.” Promoting blood circulation to remove stasis was adopted by TCM as main treatment components to manage cardiovascular complications of DM. Studies reported that this therapeutic principle may reduce blood clot formation by lowering levels of fibrinogen and D-dimer [7], as well as reducing ADP-induced platelet aggregation and plasma thrombin time [18]. However, its role in correcting traditional cardiovascular risk factors such as blood pressure, serum glucose, and lipid remains uncertain. We speculated that promoting blood circulation to remove stasis might not only correct traditional cardiovascular risk factors, but also protect type 2 diabetes patients with a YZ constitution from PAD. It should be helpful to add methods of promoting blood circulation to remove stasis into principals of conventional management to prevent PAD for patients. Moreover, if we can detect PAD-related genes of YZ constitution, investigation of potential herbal medicines to regulated related genes might be useful to protect type 2 diabetes patients with a YZ constitution from PAD. Currently, novel technique should be helpful to screen out candidate herbal medicines or natural compounds that could regulate certain genes. For example, to construct quantitative structure-activity relationship models by support vector machines and multiple linear regression method detected some natural compounds with potential efficacy in the inhibition of phosphodiesterase 4D or 5-Lipoxygenase activating protein, which are promising targets in ischemic stroke protection [19, 20].

Other independent factors which we found to be associated with PAD included age, duration of diabetes, current smoking status, and hs-CRP. Another research has also found age to be an independent risk factor for PAD [21], and the gradual increase in age in the general population in developed countries is linked to the rising prevalence of the disease. Duration of diabetes, which correlated strongly with patient's age, was also independently associated with PAD in our study, indicating that a long duration of diabetes was required for PAD to develop. Current smokers were at more than 3 times the risk for PAD compared with nonsmokers. This finding confirmed a research showing that smoking is the strongest independent risk factor associated with PAD in type 2 diabetes patients [22]. Encouraging patients to stop smoking is essential for reducing the morbidity and mortality associated with cardiovascular diseases. Finally, the inflammatory marker hs-CRP has been previously identified as a PAD risk factor [23], and the present study confirmed that it was an independent risk factor for PAD. Hs-CRP is viewed as a predictor of cardiovascular disease [24], although the exact nature of its role remains controversial.

Dyslipidemia and blood pressure have frequently been reported as risk factors for PAD [22]. In this study, TC, triglyceride, HDL cholesterol, LDL cholesterol, and DBP did not differ significantly between patients with or without PAD. We did find significant differences between the groups for SBP. However, none of these factors showed an independent association with PAD after we applied statistical adjustment to the analysis. One notable finding of our research was the fact that the influence of blood pressure and dyslipidemia was weakened by antihypertensive and lipid-lowering therapies, rendering those particular risk factors at least partially controllable. It seems possible that the combination of such therapies with the TCM method of promoting blood circulation to remove stasis might reduce the incidence of PAD in type 2 diabetic patients with a YZ constitution.

Our study was subject to two main limitations. First, to avoid subjective effects on the diagnosis of YZ constitution, we used a patient self-report questionnaire as the diagnostic tool. This meant forgoing some important TCM clinical diagnostic procedures, such as pulse diagnosis. We similarly did not use modern examination techniques and laboratory data that are usually considered when making the diagnosis of blood stasis. These deliberate omissions might have yielded lower diagnostic accuracy of YZ constitution in this study than would generally occur during clinical practice. Second, although an abnormal constitution usually occurs before a disease is diagnosed, the cross-sectional design of our study limited our ability to plot a causal relationship between YZ constitution and the development of PAD. Our results revealed only the statistical relationship between these two conditions and their associated variables.

## 5. Conclusions

Several independent factors were associated with the development of PAD in type 2 diabetic patients, namely, age, duration of diabetes, current smoking status, and hs-CRP value. In addition, we found that YZ constitution, as conceptualized by TCM, was independently associated with PAD. Our results suggested that a combination treatment for promoting blood circulation to remove stasis might prevent PAD in type 2 diabetes patients with a YZ constitution. Further investigation is needed to clarify how YZ constitution influences the development of PAD in diabetic patients.

## Figures and Tables

**Table 1:** Demographic, anthropometric, plasma, and urine biochemical traits of patients.

Characteristics	All participants	Diabetic without PAD	Diabetic with PAD	*P* value
	(*n* = 712)	(*n* = 661)	(*n* = 51)	
Age (years)	61.5 ± 10.6	61.2 ± 10.5	65.8 ± 11.7	0.003**
Male (%)	49.7	49	58.8	0.177
Diabetes duration (years)	13.1 ± 6.7	12.9 ± 6.4	16.6 ± 9.4	0.008**
Current smoking (%)	16.7	15.9	27.5	0.033*
Systolic blood pressure (mmHg)	131.7 ± 17.7	131.1 ± 17.6	139.2 ± 17.7	0.002**
Diastolic blood pressure (mmHg)	75.3 ± 10.8	75.5 ± 10.9	73.2 ± 9.5	0.145
Body height (cm)	160.2 ± 8.6	160.2 ± 8.5	160.8 ± 9.5	0.620
Body weight (kg)	65.7 ± 11.5	65.5 ± 11.5	68.3 ± 11.9	0.092
Body mass index (kg/m^2^)	25.5 ± 3.7	25.5 ± 3.7	26.3 ± 3.7	0.108
Fasting plasma glucose (mmol/L)	8.0 ± 2.4	8.0 ± 2.4	7.9 ± 2.8	0.972
Hemoglobin A1c (%)	7.9 ± 1.4	7.9 ± 1.4	8.2 ± 1.6	0.110
Total cholesterol (mmol/L)	4.8 ± 1.0	4.8 ± 1.0	5.1 ± 1.1	0.053
Triglycerides (mmol/L)	1.9 ± 1.4	1.8 ± 1.4	2.1 ± 1.4	0.141
HDL cholesterol (mmol/L)	1.2 ± 0.4	1.3 ± 0.4	1.2 ± 0.3	0.058
LDL cholesterol (mmol/L)	3.0 ± 0.9	3.0 ± 0.9	3.2 ± 0.9	0.064
hs-CRP (mg/L)	3.2 ± 9.7	2.8 ± 8.1	8.7 ± 21.0	0.049*
Creatinine (*μ*mol/L)	81.0 ± 57.2	79.2 ± 55.6	104.7 ± 71.7	0.016*
Uric acid (*μ*mol/L)	376.6 ± 105.7	373.5 ± 105.5	417.8 ± 101.0	0.004**
Normoalbuminuria (%)	58.1	60.0	33.3	<0.001**
Microalbuminuria (%)	28.4	27.7	37.3	
Macroalbuminuria (%)	13.5	12.3	29.4	
Yu-Zhi score	10.6 ± 2.7	10.5 ± 2.6	11.9 ± 3.2	0.006**
ABI ≦ 0.9 (%)	7.2			

PAD: peripheral arterial disease; HDL: high-density lipoprotein; LDL: low-density lipoprotein; hs-CRP: high-sensitivity C-reactive protein; ABI: ankle-brachial index.

Values are given either as mean ± SD or as percentages.

*P* value: comparison between diabetic patients with and without PAD; *t*-test or *χ* ^2^ test for sex, smoking, and ACR.

**Significant at *P* < 0.01.  *Significant at *P* < 0.05.

**Table 2:** Logistic regression model: odds ratios for risk factors in peripheral arterial disease (*n* = 712).

	Crude OR (95% CI)	Age- and sex-adjusted OR (95% CI)	Fully adjusted model^‡^ OR (95% CI)
Age	1.04 (1.01–1.07)**		1.04 (1.00–1.07)*
Sex			
Female	1.00 (reference)		1.00 (reference)
Male	1.49 (0.83–2.65)		1.27 (0.60–2.69)
Diabetes duration	1.07 (1.03–1.11)**	1.05 (1.02–1.10)**	1.06 (1.01–1.10)**
Current smoking			
No	1.00 (reference)	1.00 (reference)	1.00 (reference)
Yes	2.00 (1.05–3.84)*	2.77 (1.28–6.01)**	3.14 (1.33–7.39)**
Systolic blood pressure	1.02 (1.01–1.04)**	1.02 (1.00–1.04)*	1.01 (0.99–1.03)
Body mass index	1.06 (0.99–1.14)	1.08 (1.00–1.17)*	1.05 (0.96–1.14)
Hemoglobin A1C	1.16 (0.97–1.40)	1.18 (0.98–1.41)	1.02 (0.82–1.27)
Total cholesterol	1.01 (1.00-1.01)	1.01 (1.00–1.02)*	1.00 (0.98–1.03)
Triglyceride	1.00 (1.00-1.00)	1.00 (1.00-1.00)	1.00 (0.99–1.01)
HDL cholesterol	0.98 (0.96–1.00)	0.98 (0.96–1.01)	0.99 (0.96–1.03)
LDL cholesterol	1.01 (1.00-1.01)	1.01 (1.00–1.02)*	1.01 (0.98–1.03)
hs-CRP	1.33 (1.09–1.62)**	1.32 (1.10–1.58)**	1.29 (1.08–1.54)**
Creatinine	1.40 (1.00–1.96)	1.32 (0.99–1.75)	1.06 (0.76–1.47)
Uric acid	1.24 (1.07–1.43)**	1.19 (1.02–1.38)*	1.08 (0.91–1.29)
ACR			
Normoalbuminuria	1.00 (reference)	1.00 (reference)	1.00 (reference)
Microalbuminuria	2.42 (1.23–4.76)*	2.05 (1.03–4.09)*	1.40 (0.67–2.92)
Macroalbuminuria	4.31 (2.07–8.99)**	3.90 (1.86–8.20)**	1.69 (0.68–4.20)
Yu-Zhi score	1.16 (1.06–1.26)**	1.15 (1.05–1.26)**	1.10 (1.00–1.21)*

OR: odds ratio; CI: confidence interval; HDL: high-density lipoprotein; LDL: low-density lipoprotein; hs-CRP: high-sensitivity C-reactive protein; ACR: urine albumin-to-creatinine ratio.

**Significant at *P* < 0.01.  *Significant at *P* < 0.05.

^‡^Covariates in the fully adjusted model were age, sex, diabetes duration, smoking, systolic blood pressure, body mass index, hemoglobin A1C, total cholesterol, triglyceride, HDL cholesterol, LDL cholesterol, hs-CRP, creatinine, uric acid, ACR, and Yu-Zhi score.

## Appendix

Questionnaire items for measuring Yu-Zhi constitution were as follows.

1. I feel numbness in the limbs.
2. I feel chest tightness or my chest seems to be oppressed by something.
3. There is tingling pain in my body which makes me uncomfortable.
4. I have a dull sensation or pain over the lateral side (costal region) of my body.
5. Bruises appear on my skin without an apparent cause.
6. My skin gets dry, cracked, scaly, or tough.
7. My face feels dull and lustreless.
8. My lips or tongue have a dull purple color, or I find petechiae on them.

## Abbreviations

ABI: Ankle-brachial index
ACR: Albumin-to-creatinine ratio
BMI: Body mass index
BSS: Blood stasis syndrome
CI: Confidence interval
DBP: Diastolic blood pressure
DM: Diabetes mellitus
FPG: Fasting plasma glucose
HDL: High-density lipoprotein
hs-CRP: High-sensitivity C-reactive protein
LDL: Low-density lipoprotein
OR: Odds ratio
PAD: Peripheral arterial disease
SBP: Systolic blood pressure
TC: Total cholesterol
TCM: Traditional Chinese medicine
YZ: Yu-Zhi.
